# Quality Improvement Project - Initial survey of a new Mental Health of Intellectual Disabilities Service established in Ireland in January 2022

**DOI:** 10.1192/j.eurpsy.2023.2182

**Published:** 2023-07-19

**Authors:** A.-M. Curtin, B. Fitzgerald

**Affiliations:** 1 North Cork Mental Health Service, HSE, Glanmire; 2 Kerry MHID Service, Liber House, Tralee, Ireland

## Abstract

**Introduction:**

A new mental health service specialising in intellectual disabilities in Ireland was set up in January 2022. Its current compliment of staff includes is a Consultant Psychiatrist, Trainee Psychiatrist, Social Worker and Administrator. The current National Directive in Ireland is to prioritize Mental Health of Intellectual Disabilities services.

**Objectives:**

The aim of the project is to establish the current baseline level of diagnostics and interventions within the new service. Our aim is to develop this service by implementing and following the gold standard guidelines and determine what extra resources does the service need.

**Methods:**

The first fifty case notes of patients assessed by the new service were inspected. The reviewer looked for evidence of the following clinical descriptions:Diagnosis of Intellectual Disabilities and its severity; Mental Capacity; Psychiatric Diagnoses; Physical health diagnoses; Medications and evidence of a Positive Behavioural Support Plan to manage complex challenging behaviours.

**Results:**

The fifty patient audit contained 38 (76%) men and 12 (24% women) . One patient had Mild Intellectual Disabilities (ID), 39 (78%) had Moderate ID and 10 (20%) had Severe ID. All patents were very vulnerable and had limited or lacking Mental Capacity. Common diagnoses of the following were recorded in the following numbers and percentages; - Autism diagnosis 30 ( 60% ); Epilepsy 19 (38%); & Down Syndrome 9 (18%). A Formal Psychiatric diagnosis was identified in 26 (52%) of patients. Challenging Behaviour (severe and complex) was identified for 41 ( 82%) of the patients. The full breakdown of psychiatric diagnoses was ‘Psychotic illness’ – 9 (18%); Anxiety – 7(14%); Bipolar Affective Disorder 5 (10%): Depression – 4(8%); Attention Deficit Hyperactivity Disorder (ADHD) 3 (6%); Obsessive Compulsive Disorder (OCD) – 2(4%); Dementia – 2(4%): Post Traumatic Stress Disorder (PTSD) – 1 (2%); & Schizoaffective Disorder 1(2%). A Positive Behavioural Support plan (PBS) was available to support 33 (66%) of patients.

42 (84%) of patients were prescribed antipsychotic medication. 12 (24%) were prescribed more than one antipsychotic. 20 (40%) were prescribed an antipsychotic without a formally documented diagnosis of a psychotic disorder. 12 (24%).

**Conclusions:**

The results of this first survey highlight areas in which the service can be improved. The service has requested funding for a Community Nurse and a Psychologist. Psychological evaluations and Positive Behavioural Support plans are essential for people with complex challenging behaviours. A Community Nurse should assist with Health Promotion and help supervise patients requiring Depot Antipsychotic medication or Clozapine. We also plan to set up a joint clinic with the Consultant Neurologist on a regular basis.

**Disclosure of Interest:**

None Declared

